# Foreword and Dedication

**DOI:** 10.1155/MBD.1999.i

**Published:** 1999

**Authors:** Colin J. L. Lock


### **FOREWORD AND DEDICATION:** Colin J. L. Lock

Richmond, KY
8 October 1999

The 4th International Conference on Gold and Silver in Medicine was held in Milwaukee Wisconsin in June, 1998 in conjunction with the Great Lakes Regional American Chemical Society Meeting. This periodic gathering brings together chemists, pharmacologists and physicians interested in the medicinal use of these two precious metals. The participants were drawn from 8 countries and three continents. This meeting, because it is small and interdisciplinary, provides an intense interchange of ideas during the formal sessions, and perhaps more importantly, informal discussions surrounding the formal sessions.

The Conference was dedicated to the late Prof. Colin J. Lock of McMaster University. A participant in the first three Au/Ag Conferences held in Washington D.C., USA (1987), Manchester, UK (1990), and Milwaukee, USA (1994), Colin was truly one of the "Golden Boys" of bioinorganic chemistry. The contributions of his final students working on gold chemistry were summarized by his longtime collaborator and wife, Dr. Helen-Howard Lock. Colin's good humor, insight and broad grasp of bioinorganic chemistry enriched the three previous meetings and were missed by all who knew him until his untimely death in 1997.

The talks included presentations on medicinal properties of gold complexes of diphosphine and diarsine ligands, damp (dimethylaminomethylphenyl) a ligand which stabilizes gold (III) and carboranes. Other talks highlighted the structural chemistry of novel gold complexes, the role of photochemistry in characterizing gold complexes with potential activity against reactive oxygen species and the electrochemistry, disulfide interchange reactions and structures of gold thiolates. Topics of a more biological nature included the effect of gold on immune cells and a strategy for evaluating metal-based anti-tumor agents.

Metals in the same groups of the periodic table often exhibit distinctly different biochemistries: for example, the differences between sodium and potassium, which have extra and intracellular roles in neurotransmission, and the contrast of zinc, an essential element, with cadmium and mercury which are environmentally accumulated toxins. Likewise, gold and silver have very different medicinal properties. Beyond the gold compounds which are proven antiarthritic agents, others show promise against tumors, HIV and malaria. Silver is used widely as an anti-bacterial agent for treating wounds and burns in the form of silver sulfadiazene and silver treated mesh cloth, and as the metal itself in prostheses and through electrochemical activation.

Despite these differences in their applications, this fourth conference exhibited, for perhaps the first time, extensive overlap between the gold and silver presentations. A number of the more inorganic talks compared the structures and reactivity of silver complexes to their gold analogues. In addition, NMR characterization of silver-bearing proteins and circular dichroism studies of Cu, Ag and Au bound to metallothioneins, bridged the worlds of silver and gold biochemistry. Finally, impressive contributions to the biology of silver were covered in presentations on the newly discovered array of genes conferring silver resistance in bacteria and on medical value of silver-treated fabrics for wound dressings.

Finally, on behalf of all the participants, I wish to thank the Gold Institute and the Silver Institute, both of Washington, D.C., who generously provided the financial support that made the conference possible. We all look forward to the fifth gathering, which will be convened at a time and place to be decided in the near future.

C. Frank Shaw III
Professor and Chairman
Department of Chemistry
Eastern Kentucky University
Richmond, KY, 40475-3102, USA

